# Flavoaffinins, elusive cellulose-binding natural products from an anaerobic bacterium

**DOI:** 10.1101/2025.02.08.637243

**Published:** 2025-02-08

**Authors:** Duncan J. Kountz, Ruocheng Yu, Jessie H. Lee, Katherine N. Maloney, Emily P. Balskus

**Affiliations:** † Department of Chemistry and Chemical Biology, Harvard University, Cambridge, Massachusetts 02138, United States.; ‡ Department of Chemistry, Point Loma Nazarene University, San Diego, CA, 92106.; # Howard Hughes Medical Institute, Harvard University, Cambridge, Massachusetts 02138, United States

**Keywords:** Natural product, aryl polyene, cellulose, anaerobe, yellow affinity substance

## Abstract

Cellulose is the most abundant polymer on earth and plays a key role in the carbon cycle, agriculture, and human health. Many anaerobic cellulose-degrading bacteria produce uncharacterized yellow-orange, cellulose-binding pigments known as yellow affinity substances (here referred to as flavoaffinins) that are associated with efficient cellulose degradation. Here, we isolate and structurally characterize the flavoaffinins from *Clostridium* (*Hungateiclostridium*) *thermocellum*, a key workhorse for the industrial conversion of cellulosic feedstocks to ethanol. Flavoaffinins represent an unprecedented structural juxtaposition of an aryl polyene chain with a hydroxy-diene γ-lactone. We also shed light on their biosynthetic origins using stable-isotope feeding experiments. This effort lays the groundwork for understanding the biological function(s) of the flavoaffinins and expands the limited number of natural products isolated from obligately anaerobic microbes.

## Graphical Abstract

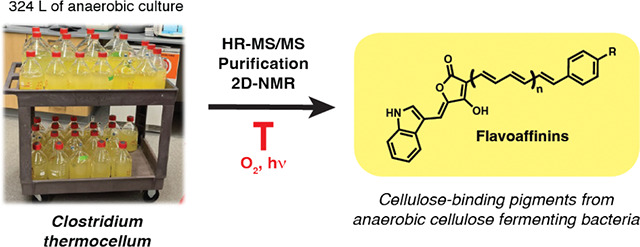

Cellulose, the primary constituent of most plant cell walls^1^, is the planet’s most abundant polymer, with 1.5 × 10^12^ tons of biomass produced annually^2^. As such, it is a major reservoir of fixed carbon, a potential renewable resource^3^, and the dominant microbial carbon source in most terrestrial ecosystems^4^. Anaerobic cellulose biodegradation to methane has a major impact on global climate change^4^, while anaerobic cellulose degradation to short-chain fatty acids in ruminant livestock indirectly sustains humanity’s food supply^5^.

The genomes of anaerobic cellulose-degrading bacteria (cellulose fermenters) are enriched in natural product biosynthetic gene clusters (BGCs)^6^. However, few natural products have been identified from anaerobic bacteria^7–11^. In 1953, McBee^12^ isolated a thermophilic cellulose fermenter (*Clostridium thermocellum*, currently classified as *H. thermocellum*^13^) that produces a water-insoluble, yellow-orange pigment when grown on cellulose^12, 14^. In 1983, Ljungdahl *et al.* noticed that this pigment binds to both cellulose and endo-glucanase, and proposed that it mediates attachment of the organism to cellulose fibrils^14^. This observation led them to name the pigment “yellow affinity substance.” Ljungdahl et al. also attempted to purify and structurally characterize yellow affinity substance^14–16^; however, all isolation attempts have failed, likely due to the oxygen and light sensitivity of the pigment.

Here, we isolate and structurally characterize the flavoaffinins, a family of structurally unique cellulose-binding polyketide pigments from *Clostridium thermocellum* that corresponds to the elusive yellow affinity substance. We also gain biosynthetic information from stable-isotopic feeding experiments, enabling future studies of the biosynthesis and biological functions of these unusual natural products.

To gain initial insights into the nature of the yellow affinity substance, we grew *C. thermocellum* DSM 1313 (see Materials and Methods) on cellulose, extracted the residual cellulose with acetone, and analyzed the extract using high-resolution tandem mass spectrometry (HR-MS/MS) with an in-line diode array UV-visible detector. This analysis revealed four major components, each with a λ_*max*_ of about 440 nm resembling the “carotenoid-like” UV-visible absorption band described by Ljungdahl *et al* (Figures 1 and S1).^14^ We observed these metabolites at *m/z* [M+H]^+^ 424.15, 450.17, 408.16, and 434.18, consistent with molecular formulae C_27_H_21_NO_4_, C_29_H_23_NO_4_, C_27_H_21_NO_3_, and C_29_H_23_NO_3_ (Table S1 and Figures S2 and S3). MS^2^ analysis revealed that each analyte produces prominent fragment ions with *m/z* 240.07 (C_14_H_10_NO_3_^+^) and 158.06 (C_10_H_8_NO^+^) (Figure S4). We named these metabolites flavoaffinins 423, 449, 407, and 433 in accord with the nominal masses of the neutral molecules.

We next optimized culturing and purification conditions to obtain sufficient quantities of flavoaffinins for characterization (Figure 2). As previously reported, we found that flavoaffinin production is induced by cellulose^14–16^. However, supplementation of cultures with 5 mM sodium acetate also induced flavoaffinin production during growth on cellobiose. Use of cellobiose permitted extraction of the flavoaffinins from the cell pellet with acetone. We used the flavoaffinins’ strong affinity for binding a microcrystalline cellulose affinity column later in the purification. We confirmed that flavoaffinins readily degrade under both light and oxygen, forming many unidentified species (Figure S5). Thus, we carried out the purification under red light and/or in an anaerobic chamber. After developing a workflow that maintained the structural integrity of the flavoaffinins, we obtained 3.9 mg of flavoaffinin 407 and 4.0 mg of flavoaffinin 449 from 324 L of *C. thermocellum* culture for structure determination.

Flavoaffinin 407 (**1**) (Figure 3 Table S2) was observed in HR-ESI-MS at *m/z* 408.1600, consistent with a molecular formula of C_27_H_21_NO_3_ (calcd 408.1605) and 18 degrees of unsaturation. The ^13^C NMR spectrum included 17 methine and 8 non-protonated carbon signals at chemical shifts ranging from δ_c_ 97.9 to 170.9. COSY/TOCSY revealed four spin systems, including an aromatic methine proton at δ_H_ 7.95 (H-2) coupled to an exchangeable proton at δ_H_ 10.70 (H-1), a disubstituted benzene ring (H-5 through H-8), a phenyl group (H-24 through H-26), and a conjugated polyene chain (H-15 through H-22). HMBC correlations from H-2 to C-3, C-4 and C-9; from H-5 and H-7 to C-9; and from H-6 and H-8 to C-4 permitted assembly of the first two spin systems into an indole ring system substituted at the 3-position. At the other end of the molecule, HMBC correlations from H-21 to C-23, from H-22 to C-24, from H-24 to C-22 and from H-25 to C-23 connected the phenyl group to the aryl polyene chain. The dearth of proton-bearing carbons made elucidation of the remainder of the molecule challenging. However, key HMBC correlations from H-10 to C-4, C-11 and C-12 and from H-15 to C-12, C-13 and C-14 led us to propose the hydroxy-diene-γ-lactone moiety shown. The all-trans configuration of the polyene was determined based on the 3-bond proton coupling constants (*J*_*15,16*_ = 15.4 Hz; *J*_*16,17*_ = 11.5 Hz; *J*_*17,18*_ = 14.6 Hz; *J*_*18,19*_ = 11.4 Hz; *J*_*19,20*_ = 15.0 Hz; *J*_*21,22*_ = 15.6 Hz), and supported by key NOESY correlations between H-15 and H-17; H-16 and H-18; H-19 and H-21; and H-21 and H-24. A NOESY correlation between H-5 and H-10 suggested a *s*-cis conformation for the C-3/C-10 bond. We assume the *Z*-configuration for the C-10/C-11 double bond for steric reasons. The conformation of the C-14/C-15 bond is not resolved by our data (Table S2).

NMR data for flavoaffinin 449 (**4**) (Table S3) were similar to those of **1**, leading us to propose the same general structure, consisting of a ‘head group’ composed of an indole linked to a hydroxy-diene-γ-lactone moiety, connected via an aryl polyene chain to a ‘tail’ group. Apparent degradation of the flavoaffinin 449 sample during anoxic storage at −70 °C may be attributed to partial oxidation (by disproportionation) of the phenolic hydroxyl at C-26 followed by tautomerization through the C-12 enol as suggested by impaired resolution (Figures S21 and S22) of the signals in the polyene region of the spectrum. Nonetheless, we could tentatively assign the tail group as a *para*-substituted benzene ring (See NMR data in Table S3) on the basis of doublets at δ_H_ 7.29 (H-24) and 6.78 (H-25). The HR-MS/MS data discussed below suggests that the substituent is a *para*-hydroxy group.

The NMR-based structural assignments for flavoaffinin 407 and 449 are corroborated by HR-MS/MS data, which further permitted us to infer the structures of flavoaffinins 423 (**2**) and 433 (**3**) (Figure S4). All four flavoaffinin congeners produce a C_14_H_10_NO_3_^+^ fragment ion (A4, see Figure 3D) corresponding to the head group. The presence of a monosubstituted indole ring was consistent with the production of indolium (C_8_H_8_N^+^, A1) and quinolinium (C_9_H_8_N^+^, A2) ions by all four congeners. A polyene chain extending from the head group was supported by inspection of low-abundance ions, which revealed successive extension of the head group ion by multiple acetylene (C_2_H_2_) units (Figure S4).

Flavoaffinins 423 and 449 each produced abundant “tail group” hydroxytropylium ions (C_7_H_7_O^+^) suggestive of a hydrocarbon-substituted phenol. In the flavoaffinin 407 and 433 MS^2^ spectra, the hydroxytropylium was not present, having been replaced by unsubstituted tropylium ions (C_7_H_7_^+^), suggesting a hydrocarbon substituted benzene ring in these congeners. In total, the HR-MS/MS data support the proposed structures of flavoaffinins 407 and 449, and suggest likely structures of the less abundant flavoaffinins 423 and 433.

The flavoaffinins are unusual bacterial natural products. While hydroxy-diene-γ-lactones are common in plant and fungal natural products, they are rare in bacteria. The core structure of the flavoaffinins resembles the aspulvinone^17,18^ pigments produced by the conidia of *Aspergillus* spp.; in both cases a hydroxy-diene-γ-lactone moiety is situated between two aryl groups. In the aspulvinones, these aryl groups are phenyl or phenyl derivatives, rather than the indole of the flavoaffinins (Figure S35). Moreover, the non-indole aryl group of the flavoaffinins is separated from the central lactone by a polyene chain, which is unprecedented. Aryl polyenes are well-known polyketide natural products that are antioxidants and/or photo-protectants and are often covalently-incorporated into the bacterial cell wall^19–21^. In most aryl polyenes, the terminus of the polyene chain opposite the aryl group is a carboxylic acid or primary alcohol. Although putative aryl polyene BGCs are ubiquitous in sequenced genomes^22^, the flavoaffinins are (to our knowledge) the first aryl polyenes isolated from an obligately anaerobic bacterium. The flavoaffinin structures also provide potential insights into the origins of their cellulose-binding activity. Indole and indole-derived dyes are known to bind cellulose^23,24^. Moreover, many cellulose-binding proteins interact with this insoluble substrate via the indole rings of multiple tryptophan residues that are often critical for attachment^25–26^. These observations suggest the flavoaffinin indole moiety may be important for cellulose binding.

We next investigated the biosynthesis of the flavoaffinins. To test the hypothesis that the indole ring was derived from l-tryptophan, we performed stable isotopologue feeding experiments in *C. thermocellum* grown on cellobiose + acetate. Feeding *C. thermocellum* U-^13^C-l-tryptophan resulted in incorporation of all eleven carbons of tryptophan into each of the four flavoaffinin congeners (Figure 4B and Figure S36B). Next, we assessed the origin of the terminal aryl group. As *C. thermocellum* lacks orthologs of genes involved in benzoate or *p*-hydroxybenzoate biosynthesis, we hypothesized that the aryl group would derive from l-phenylalanine (flavoaffinins 407 and 433) or l-tyrosine (flavoaffinins 423 and 449). Feeding experiments demonstrated incorporation of ring-D_5_-l-phenylalanine into flavoaffinins 407 and 433 (Figure 4C), and ring-D_4_-l-tyrosine into flavoaffinins 423 and 449 (Figure S36C). Finally, we hypothesized that the polyene chain would derive from acetate via the action of polyketide synthase (PKS) machinery. Feeding U-^13^C-acetate resulted in incorporation of up to three and four detected ketide units into flavoaffinins 407 and 449, respectively (Figure 4D and Figure S36D).

The results of these feeding experiments allowed us to search for putative flavoaffinin BGCs in the *C. thermocellum* genome. In particular, the labeling pattern observed when feeding acetate suggests the potential involvement of PKS biosynthetic machinery, as is the case with all other aryl polyenes^20–22^. *C. thermocellum* DSM 1313 possesses a single PKS BGC (provisionally named the *faf* gene cluster) that is a strong candidate for flavoaffinin biosynthesis. This BGC encodes a type I PKS (FafF) with a domain architecture consistent with aryl polyene biosynthesis, along with additional biosynthetic enzymes that could account for flavoaffinin production (Figure S37). Though thousands of polyketides have been described from aerobic and facultatively anerobic bacteria, only a handful have been isolated from obligately anaerobic bacteria, making the flavoaffinins important additions to this class of natural products^7–10^.

Anaerobic cellulolytic bacteria are important industrial, ecological, agricultural, and environmental microbes. By elucidating the chemical structures of the enigmatic flavoaffinins, we now set the stage for investigating the biosynthesis and physiological functions of these intriguing natural products. Our findings also highlight anaerobic cellulolytic bacteria as an exciting source for the discovery of bioactive small molecules.

## Supplementary Material

Supplement 1

## Figures and Tables

**Figure 1:**
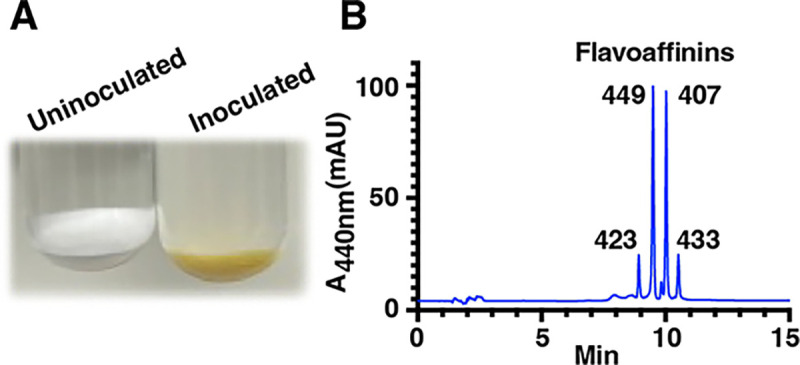
*C. thermocellum* “yellow affinity substance” is composed of four major cellulose-binding components (flavoaffinins). (A) Cellulose sediments from uninoculated (left) and inoculated (right) growth media following incubation at 60 °C for three days showing the flavoaffinins adhering to the cellulose. (B) HPLC analysis of acetone extracts of cellulose sediment from a *C. thermocellum* culture. Peaks corresponding to flavoaffinins 423, 449, 407, and 433 are indicated.

**Figure 2:**
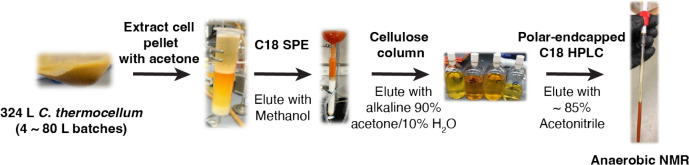
Flavoaffinin purification process. The purification could be conducted under an ambient atmosphere, but exposure to light was prevented whenever possible. Even in the dark and at 4 °C, the preparations lost color under ambient atmosphere over the course of a few days. Anoxic NMR in a vacuum tube was essential for compound stability over the course of data acquisition.

**Figure 3:**
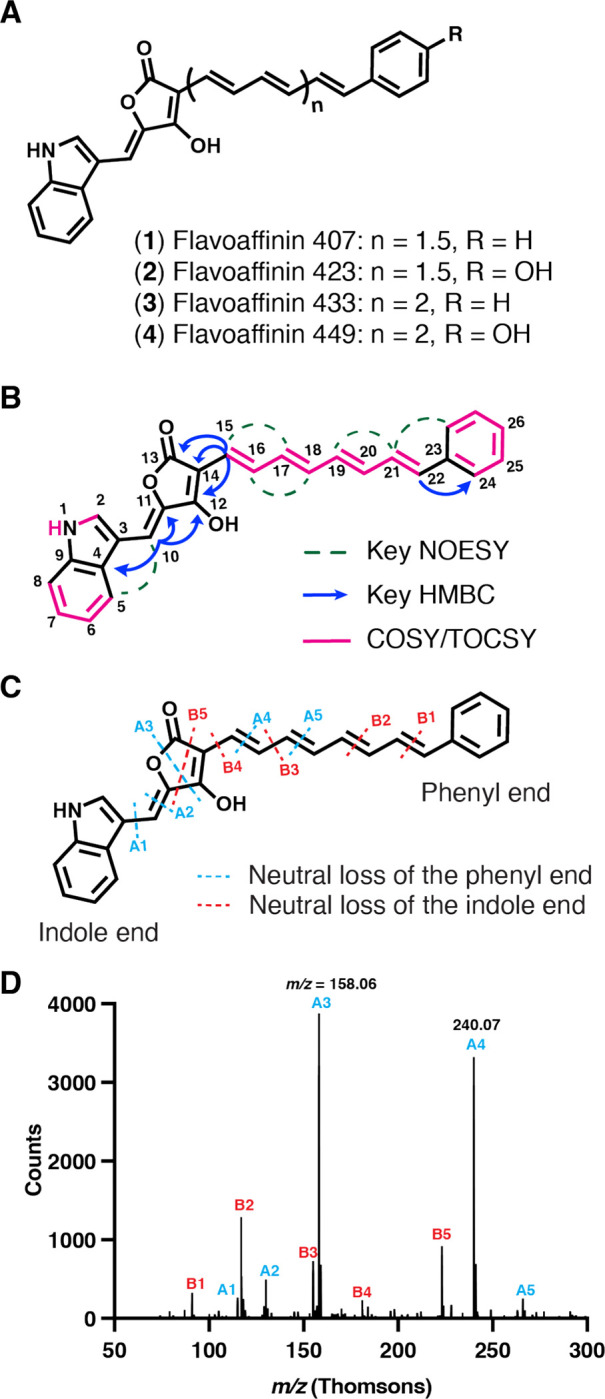
Structure elucidation of flavoaffinin 407 by NMR and HR-LC-MS/MS. (A) Proposed structures of flavoaffinins 407, 423, 433, and 449. (B) Key NOESY, HMBC, and COSY/TOCSY correlations observed for flavoaffinin 407. (C) HR-CID-MS^2^ fragmentation diagram for flavoaffinin 407 ([M + H]^+^ precursor ion). Blue and red lines indicate formation of fragment ions with neutral loss of the portion of the precursor ion connected to the phenyl group or indole ring, respectively. (D) HR-CID-MS^2^ of flavoaffinin 407 ([M + H]^+^ precursor ion.) Each peak is labeled with the name of the corresponding fragment ion given in panel C.

**Figure 4:**
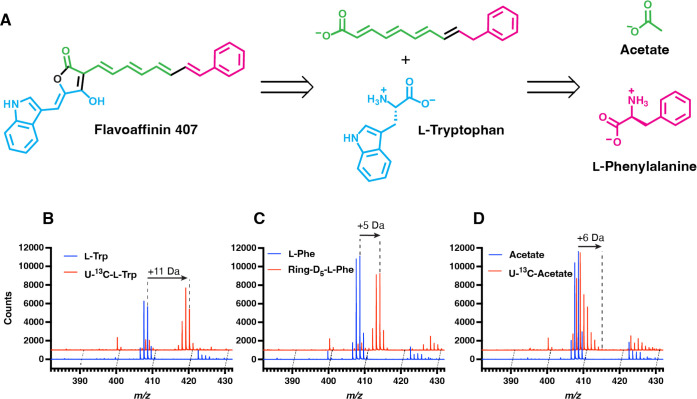
Identification of flavoaffinin biosynthetic precursors. (A) Biosynthetic hypothesis for flavoaffinin 407. (B) HR-LC-MS spectra of *C. thermocellum* fed either 0.25 mM unlabeled l-tryptophan (l-Trp) or 0.25 mM uniformly ^13^C-labeled l-tryptophan (U-^13^C-l-Trp). (C) HR-LC-MS spectra of *C. thermocellum* fed either 0.75 mM unlabeled L-phenylalanine (l-Phe) or 0.75 mM ring-perdeuterated l-phenylalanine (Ring-D_5_-l-Phe). (D) HR-LC-MS spectra of *C. thermocellum* fed either 10 mM unlabeled sodium acetate (Acetate) or 10 mM uniformly ^13^C-labeled sodium acetate (U-^13^C-Acetate). For the ESI-positive mode mass spectra in (B)-(D) each flavoaffinin analyte was ionized by multiple pathways, including as a proton adduct, as a radical cation, and following in-source dehydrogenation, as previously described in ESI-MS of other polyenes^28^.

## ABBREVIATIONS

MS: mass spectrometry
LC, HPLC: high-performance liquid chromatography liquid chromatography
HR-MS/MS: high-resolution tandem mass spectrometry
UV: ultra-violet
NMR: nuclear magnetic resonance
COSY: ^1^H,^1^H-correlation spectroscopy
TOCSY: ^1^H,^1^H-total correlation spectroscopy
NOESY: nuclear Overhauser effect spectroscopy
HSQC: ^1^H,^13^C-heteronuclear single-quantum correlation spectroscopy
HMBC: ^1^H,^13^C-heteronuclear multiple-bond correlation spectroscopy
BGC: biosynthetic gene cluster
PKS: polyketide synthase
ESI: electrospray ionization
